# *Notes from the Field:* Outbreak of Campylobacter jejuni Associated with Consuming Undercooked Chicken Liver Mousse — Clark County, Washington, 2016

**DOI:** 10.15585/mmwr.mm6638a4

**Published:** 2017-09-29

**Authors:** Derel Glashower, Jennifer Snyder, Diane Welch, Shannon McCarthy

**Affiliations:** 1 Clark County Public Health, Vancouver, Washington.

On July 13, 2016, Clark County (Washington) Public Health (CCPH) received a report of diarrheal illness in four of seven members of a single party who dined at a local restaurant on July 6, 2016. The report was received through an online/telephone system for reporting food service–associated illness complaints. Members of the five households in the party reported that their only shared exposure was the restaurant meal. CCPH ordered closure of the restaurant kitchen on July 13, 2016, and began an investigation to identify the source of diarrheal illness and implement additional control measures.

CCPH defined a probable case of restaurant-associated illness as diarrhea lasting >2 days in any restaurant guest or staff member with illness onset from July 1, 2016, to July 23, 2016. After *Campylobacter jejuni* was cultured from stool specimens submitted by three ill members of the dining party, a confirmed case was defined as culture evidence of *C. jejuni* infection in any restaurant guest or staff member with onset of diarrheal illness during the same period. Five cases (three confirmed and two probable) were identified, four in restaurant guests and one in a food worker; patient age ranged from 27–46 years; three patients were female.

CCPH conducted a case-control study involving 28 menu items, using 14 non-ill dining companions and restaurant staff members as controls. Consumption of two menu items, chicken liver mousse (odds ratio [OR] = 36.1, 95% confidence interval [CI] = 1.58–828.9), and grilled romaine hearts (OR = 18, 95% CI = 1.19–271.5) were associated with case status. Because of the higher odds ratio of chicken liver mousse and previous *Campylobacter* outbreaks associated with chicken livers (*1*,*2*), the investigation focused on the mousse.

During an inspection on July 15, the sous-chef solely responsible for preparing the chicken liver mousse demonstrated preparation to the CCPH food safety inspector, who observed that the sous-chef used the appearance of the livers alone to determine whether they were fully cooked. Final internal cook temperature of the largest liver measured by the inspector was <130°F (54°C), below the minimum 165°F (74°C) internal temperature deemed necessary by the Food and Drug Administration to eliminate food safety hazards (*3*). Because raw chicken parts are not required to be free of *Campylobacter* (*4*), and the bacteria might be present on the surface of 77% of retail chicken livers (*5*), CCPH immediately addressed undercooking of the livers.

One patient stool specimen isolate was available for typing by pulsed-field gel electrophoresis (PFGE). The PFGE pattern from this isolate was indistinguishable from those obtained from two chicken liver samples collected in a 2014 campylobacteriosis outbreak in Oregon (*1*). Chicken livers associated with both the 2014 outbreak and with this outbreak were supplied by the same company. Chicken livers from the lot served at the restaurant on the day of the implicated meal were no longer available; therefore, the U.S. Department of Agriculture could not pursue testing of chicken liver samples.

Among published *C. jejuni* outbreaks associated with undercooked chicken livers, this outbreak report is the second from the Pacific Northwest (*1*), and the first in the United States initially reported through an illness complaint system. Because CCPH does not actively investigate *Campylobacter* cases in persons aged >5 years, and because *Campylobacter* PFGE is not routinely conducted in Washington, this outbreak would have likely gone undetected if not for the illness complaint system, demonstrating the value of illness complaint investigations to identify outbreaks and mitigate public health risks.
